# Editorial: Innovative vaccine development strategies for parasitic diseases

**DOI:** 10.3389/fimmu.2026.1903207

**Published:** 2026-06-26

**Authors:** Qingbin Guo, Xinming Tang, Nishith Gupta

**Affiliations:** 1 Key Laboratory of Animal Biosafety Risk Prevention and Control (North) and Key Laboratory of Veterinary Biological Products and Chemical Drugs of MARA, Institute of Animal Science, Chinese Academy of Agricultural Sciences, Beijing, China; 2 Intracellular Parasite Education and Research Labs (iPEARL), Department of Biological Sciences, Birla Institute of Technology and Science, Pilani (BITS Pilani), Hyderabad, India

**Keywords:** antigen, delivery system, immune response, parasite, vaccine

## 1 Introductions

Parasitic diseases remain among the most formidable challenges in global public health and veterinary medicine. Driven by complex multistage life cycles and sophisticated immune evasion strategies, these pathogens inflict a staggering socioeconomic toll. The numbers underscore the urgency: in 2023 alone, malaria accounted for an estimated 263 million clinical cases and nearly 597,000 deaths. Neglected tropical diseases, such as leishmaniasis, Chagas disease, and cystic echinococcosis, continue to devastate marginalized populations, while veterinary infections like coccidiosis threaten global livestock productivity. Compounding these biological challenges, the escalating crisis of drug resistance is rapidly eroding the efficacy of mainstay therapies. Consequently, the development of prophylactic and therapeutic vaccines represents the most sustainable strategy for long-term parasitic disease control. Historically, traditional vaccine approaches, relying on immunodominant surface antigens or whole-killed organisms, have struggled to induce durable and cross-strain immunity. The thematic Research Topic, *Innovative Vaccine Development Strategies for Parasitic Diseases*, consolidates ten contributions, including nine original research articles and one review. By shifting toward rationally designed next-generation vaccines, multi-omics target discovery, and advanced delivery platforms, these studies offer an accessible roadmap for translating laboratory breakthroughs into clinical efficacy.

## 2 Finding concealed antigens and glycoproteins

Historically, antigen selection has heavily favored structurally accessible surface proteins. However, these molecules are under the most intense evolutionary pressure to mutate and evade host immunity. To circumvent this, contemporary strategies are pivoting toward concealed antigens, internal functional proteins shielded from the host immune system during natural infection. In this Research Topic, Rechsteiner et al. isolated gut-associated glycoproteins from the liver fluke *Fasciola hepatica*. Using monoclonal antibodies against these concealed targets, they successfully stunted the fluke’s motility, fecundity, and active secretion mechanisms. The team identified 36 highly conserved protein candidates that could serve as stable, evasion-resistant vaccine targets. As critical as the choice of target is its molecular architecture. Parasites extensively utilize post-translational modifications, particularly N-linked glycosylation, to orchestrate their surface proteins. Analyzing the H11 glycosylated aminopeptidase of the *Haemonchus contortus*, Liu et al. mapped 19 precise N-glycosylation sites and 31 unique N-glycan structures. They discovered that protective host antibodies selectively target these specific intact glycopeptides rather than the underlying protein. This finding underscores a pivotal principle: future synthetic vaccines must employ advanced eukaryotic expression systems capable of faithfully replicating these complex parasite-specific glycan structures to achieve authentic protective efficacy.

## 3 Reading the natural immunopeptidome

Vaccines designed to elicit robust T-cell immunity often rely on computer algorithms to predict which peptide fragments will bind to host immune cells. Although cost-effective, these in silico approaches frequently prove inadequate because biochemical binding potential does not guarantee natural processing and presentation during live infection. In this context, Vrij et al. circumvented computational prediction entirely by directly sampling skin biopsies from patients infected with *Leishmania aethiopica*. Using mass spectrometry, they identified 580 naturally presented epitopes. Strikingly, standard predictive algorithms missed 20%–70% of these physiologically relevant targets while simultaneously generating millions of false-positive binders. Jiang et al. proved the power of utilizing naturally presented epitopes in the development of the FL46 multi-epitope vaccine against *Echinococcus granulosus*. By combining empirically validated, HLA-bound human epitopes, the vaccine generated long-lasting cellular and humoral immune responses in mice. Vaccinated subjects achieved a nearly 59% suppression of parasitic cysts alongside significant reductions in hepatic fibrosis.

## 4 Smarter delivery and formulation platforms

The intrinsic immunogenicity of a candidate antigen is seldom sufficient alone. The delivery platform critically dictates the magnitude and durability of the host immune responses. To address the waning antibody titers observed with single-antigen malaria vaccines, Turan et al. engineered bivalent virus-like particles (VLPs) simultaneously displaying two distinct *Plasmodium falciparum* antigens: CSP and SPECT-1. These multivalent constructs generated high-avidity antibodies without immune interference between antigens, yielding protective efficacy comparable to the benchmark R21/Matrix-M vaccine. For Chagas disease, Antonoglou et al. reported that a chimeric DNA vaccine (CruSEG) delivered orally via an attenuated *Salmonella* vector drove potent and systemic cellular immunity. Alternatively, the recombinant protein version skewed the immune responses toward humoral protection, but both dramatically suppressed chronic parasite burdens. One of the greatest hurdles for current vaccines such as R21 is the demanding multi-dose regimen required to maintain protection. Mukhopadhyay et al. successfully adapted immune complexes (ICs) technology to circumvent this limitation. By pre-complexing the R21 vaccine antigen with its specific neutralizing antibody, they optimized uptake by antigen-presenting cells. This formulation achieved robust 100% sterile protection in mice, using a streamlined two-dose regimen administered subcutaneously.

## 5 Advanced evaluation frameworks

The translation of highly sophisticated vaccine constructs from controlled murine models to verifiable human efficacy requires rigorous clinical evaluation frameworks. Two distinct studies in this thematic collection focus explicitly on how we predict and define vaccine-induced immunity directly within human populations. Controlled Human Malaria Infection (CHMI), in which healthy volunteers are deliberately and safely infected, has become an indispensable tool for predicting vaccine field efficacy and mitigating the risk of costly Phase III trials. Ogwang et al. reviewed the history, rationale, and applications of falciparum malaria CHMI models, emphasizing lessons derived from both naive and endemic populations. When combined with high-resolution genomic tools, CHMI provides unparalleled insights into vaccine-induced immunity. Utilizing an ultra-high-density peptide microarray comprising over 638,000 unique peptides, Friedman-Klabanoff et al. probed sera from volunteers infected with distinct malaria strains. Although the overall intensity of the antibody response was broadly comparable between cohorts, high-resolution mapping revealed critical differences in the breadth of serological cross-reactivity across approximately 10% of the tested proteome. Crucially, the analysis identified a highly cross-reactive, non-asparagine-rich epitope located on the Early Transcribed Membrane Protein 10.2 (ETRAMP10.2). Given that over 95% of the variant peptides representing this specific ETRAMP10.2 epitope were universally recognized by immune sera from both infection cohorts, this antigen represents a prime, highly stable, and previously underutilized target for next-generation, cross-strain malaria vaccines.

## 6 Expanding the subunit paradigm in veterinary medicine

Veterinary parasitology fundamentally underpins global agricultural economics and animal welfare. Avian coccidiosis, caused by *Eimeria* protozoa, inflicts devastating losses on the poultry industry. Addressing the limitations of current live-attenuated vaccines, researchers have targeted dense granule proteins (GRAs). Rigorous sequence variation analysis across global field isolates revealed that putative targets like *Et*GRA9 and *Et*GRA12a are under strict, intense purifying selection. This genomic signature indicates essential functional importance and long-term structural stability, rendering these proteins ideal subunit vaccine candidates with reduced susceptibility to mutational escape. Leveraging these biological properties, Sánchez-Arsuaga et al. produced recombinant *Et*GRA9 and evaluated its efficacy via *in vivo* challenge trials. Immunizing commercial chickens with the recombinant *Et*GRA9 antigen resulted in an impressive 85.7% reduction in parasite burden, statistically comparable to established standard vaccine candidates. By establishing dense granule proteins as highly protective and stable immunogens, this foundational work significantly expands the repertoire of viable targets for engineering cost-effective recombinant poultry vaccines.

## 7 Conclusion

In summary, these studies collectively represent significant progress toward effective vaccines against parasitic diseases and establish a strong foundation for future development. By focusing on hidden glycoproteins and naturally presented immunopeptidomes rather than relying purely on computer predictions, researchers are systematically overcoming historical roadblocks. These advances, combined with smarter delivery vehicles such as multivalent VLPs, ICs and validated through high-resolution clinical frameworks like CHMI, are anticipated to accelerate the bench-to-clinic translation of next-generation antiparasitic vaccines, ultimately alleviating the global burden of parasitic diseases.

